# Complete Genome Sequences of Two Genetically Distinct Variants of Porcine Epidemic Diarrhea Virus in the Eastern Region of Thailand

**DOI:** 10.1128/genomeA.00634-15

**Published:** 2015-06-25

**Authors:** Thaniwan Cheun-Arom, Gun Temeeyasen, Anchalee Srijangwad, Thitima Tripipat, Suphattra Sangmalee, Dam Thi Vui, Taksina Chuanasa, Angkana Tantituvanont, Dachrit Nilubol

**Affiliations:** a Department of Veterinary Microbiology, Faculty of Veterinary Science, Bangkok, Thailand; b Department of Pharmacognosy and Pharmaceutical Botany, Faculty of Pharmaceutical Sciences, Chulalongkorn University, Bangkok, Thailand; c Virology Section, National Center for Veterinary Diagnosis, Department of Animal Health, Hanoi, Vietnam; d Department of Pharmaceutics and Industrial Pharmacy, Faculty of Pharmaceutical Sciences, Chulalongkorn University, Bangkok, Thailand

## Abstract

Porcine epidemic diarrhea virus (PEDV) has continued to cause sporadic outbreaks in Thailand since 2007. Previously, PEDV in Thailand was a new variant containing an insertion and deletion in the spike gene. Herein, full-length genome sequences are reported for two variants of PEDV isolates from pigs displaying diarrhea in Thailand.

## GENOME ANNOUNCEMENT

Porcine epidemic diarrhea (PED) is a devastating enteric disease (1) caused by the PED virus (PEDV), an RNA virus in the genus *Alphacoronavirus*, family *Coronaviridae*, order *Nidovirales* (2). Currently, PEDV poses a threat to the swine industry worldwide (3–8). In Thailand, PED has been observed since 2007 (8). However, between 2013 and 2014, several herds in the eastern region of Thailand experienced recurring outbreaks of PED. This investigation is based on a complete spike (S) gene sequence revealing two genetically distinct variants, for which the whole genome sequences are reported.

Two PEDV variants, designated CBR1 and EAS1, were isolated from 3-day-old pigs with PED from farms in the eastern region of Thailand using the Vero cell line (9). Total RNA was extracted from culture supernatant. Twelve overlapping regions of each genome were amplified, cloned in pGEM-T easy vector (Promega, USA), and sequenced in both directions in triplicate per the previously reported protocol (10). The 5′-terminal sequences were determined by 5′ rapid amplification of cDNA ends (RACE).

Two PEDV isolates, CBR1 and EAS1, are 28,035 and 28,033 nucleotides (nt) in length, respectively. The isolates are 96.2% and 91.8% identical at the nucleotide and amino acid levels, respectively. Genome organization resembles that of other PEDV genomes (2, 11), with gene order 5′-ORF1a/1b-S-ORF3-envelop (E)-membrane (M)-nucleocapsid (N)-3′. Open reading frame a/1b (ORF1a/1b) encodes nonstructural proteins, which were subdivided to 1a and 1b, with sizes of 12,309 nt (nt 293 to 12601 and 297 to 12605) and 8,037 nt (nt 12601 to 20637 and 12605 to 20641), respectively. EAS1 is 6 nt shorter in the S gene than CBR1. The other 3 structural proteins are equal in size, 231, 681, and 1,326 nt, respectively. An accessory protein, ORF3, of both isolates encodes a similar size of 675 nt (CBR1, nt 24791 to 25465; EAS1, nt 24789 to 25463).

A comparison of PEDV sequences available in GenBank demonstrated that CBR1 shares a high similarity (98.3% to 98.7% and 96.3% to 97.0% at the nucleotide and amino acid levels, respectively) with more recent isolates from China (12). In contrast, EAS1 shares a high similarity with LZC, SM98, and CV777 (99.1% to 99.5% and 98.3% to 98.8% at the nucleotide and amino acid levels, respectively). Compared to CBR1, EAS1 has 2 insertions of 4 (^56^GENQ^59^) and 1 (^140^N) amino acids at positions 56 to 59 and 140 and 2 deletions of 2 (^160^DG^161^) and 1 (^1199^Y) amino acids at positions 160 to 161 and 1199. The insertions and deletions are located in the hypervariable domain in the N terminus of the S1 region.

A comparison to complete spike gene sequences available in GenBank demonstrated that CBR1 shared 94.2% to 98.5% and 92.2% to 98.0% nucleotide sequence and amino acid similarity with the prevalent PEDV variant in China and Thailand (8), In contrast, EAS1 has a close relationship with CV7777, LZC, and SM98 vaccine strains, sharing 98.4% to 98.9% and 96.6% to 97.5% nucleotide sequence and amino acid similarity and is genetically distinct from the prevalent PEDV variant in Thailand. These results suggest that EAS1 is a novel isolate in Thailand.

The results of the whole-genome sequence demonstrated that novel PEDV variants are circulating in Thailand. Studies investigating the molecular epidemiology, prevalence of either variant, and evolution of PEDV in Thailand are urgently required.

### Nucleotide sequence accession numbers.

The complete genome sequences of CBR1 and EAS1 have been deposited in GenBank under the accession numbers KR610993 and KR610991, respectively.
